# Detecting Changes in Retinal Function: Analysis with Non-Stationary *Weibull* Error Regression and Spatial Enhancement (*ANSWERS*)

**DOI:** 10.1371/journal.pone.0085654

**Published:** 2014-01-17

**Authors:** Haogang Zhu, Richard A. Russell, Luke J. Saunders, Stefano Ceccon, David F. Garway-Heath, David P. Crabb

**Affiliations:** 1 School of Health Sciences, City University London, London, United Kingdom; 2 Institute of Ophthalmology, University College London, London, United Kingdom; 3 National Institute for Health Research Biomedical Research Centre for Ophthalmology, Moorfields Eye Hospital NHS Foundation Trust and UCL Institute of Ophthalmology, London, United Kingdom; Dalhousie University, Canada

## Abstract

Visual fields measured with standard automated perimetry are a benchmark test for determining retinal function in ocular pathologies such as glaucoma. Their monitoring over time is crucial in detecting change in disease course and, therefore, in prompting clinical intervention and defining endpoints in clinical trials of new therapies. However, conventional change detection methods do not take into account non-stationary measurement variability or spatial correlation present in these measures. An inferential statistical model, denoted ‘Analysis with Non-Stationary *Weibull* Error Regression and Spatial enhancement’ (*ANSWERS*), was proposed. In contrast to commonly used ordinary linear regression models, which assume normally distributed errors, *ANSWERS* incorporates non-stationary variability modelled as a mixture of *Weibull* distributions. Spatial correlation of measurements was also included into the model using a Bayesian framework. It was evaluated using a large dataset of visual field measurements acquired from electronic health records, and was compared with other widely used methods for detecting deterioration in retinal function. *ANSWERS* was able to detect deterioration significantly earlier than conventional methods, at matched false positive rates. Statistical sensitivity in detecting deterioration was also significantly better, especially in short time series. Furthermore, the spatial correlation utilised in *ANSWERS* was shown to improve the ability to detect deterioration, compared to equivalent models without spatial correlation, especially in short follow-up series. *ANSWERS* is a new efficient method for detecting changes in retinal function. It allows for better detection of change, more efficient endpoints and can potentially shorten the time in clinical trials for new therapies.

## Background and Significance

**Figure 1:**
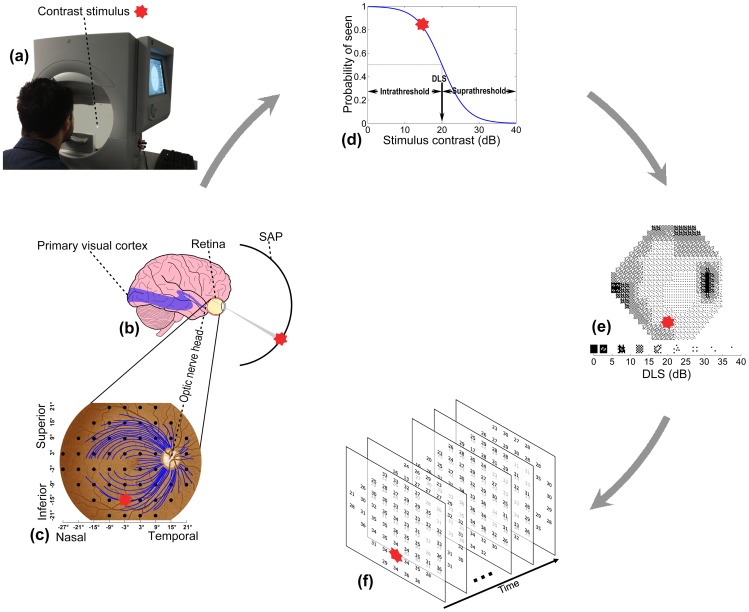
Visual field measured by standard automated perimetry (SAP). (a) Contrast stimulus from SAP is projected on different locations of retina. The response from subject is captured when the stimulus is perceived. (b) SAP assesses differential light sensitivity (DLS) of the retina and corresponding visual pathway. (c) DLSs are measured at various locations (dots) on the retina. The point (0°,0°) indicates central vision that corresponds to the fovea on the retina. Optic nerve head is the anatomical blind spot. The test locations are not only correlated to their neighbours but also by the optic nerve fibres (some of which are shown as blue curves) passing through them. The whole visual field can be divided into superior and inferior hemifields on vertical and nasal and temporal regions on horizontal. (d) The DLS at a location on the retina is derived at the 50% probability of the visual system responding to a contrast stimulus and is related to the biological response to light of relay neurones in the visual pathway. (e) The DLS is measured in log scale, which in Humphrey Field Analyzer (Carl Zeiss Meditec Inc, Dublin, CA, USA) is calculated as dB = 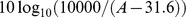 where 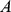 is the luminance of the stimulus in apostilbs and 31.6 apostilbs is the background luminance. The DLS ranges between 0 dB (high contrast stimulus, blindness) and around 35 dB (low contrast stimulus, healthy) and is displayed as a conventional gray-scale plot. Darker shading represents lower DLS. (f) Measurements of DLS over time form a complex spatial-temporal time series.

In recent years great strides have been made in understanding ocular diseases in the research laboratory and *in vivo*, leading to the elucidation of neuro-regenerative processes and even reversing blindness in some conditions.[1]–[4] The retina, uniquely, is an accessible and directly visible extension of the brain and, therefore, retinal research is becoming a focus for unravelling the complexity of other neurological changes such as those observed in Alzheimer's disease,[5], [6] multiple sclerosis [7], [8] and Gaucher disease.[9] The primary goal in the management of most eye conditions is preservation or improvement in visual function. An established reference test for visual function, namely the visual field, is Standard Automated Perimetry (SAP; Figure 1a). SAP measures the differential light sensitivity (DLS), across a person's retina and the corresponding visual pathway (Figure 1b,c). Unfortunately, development of computational and statistical methods for analysing data from SAP has not kept pace with the advances in other aspects of eye-related research. Nevertheless, SAP is used extensively in eye and neurology clinics, especially in the detection and management of glaucoma, a group of chronic optic neuropathies causing progressive loss of retinal ganglion cells and their axons and resulting in loss of retinal function. This disease represents a large global health problem with about 80 million people expected to be affected by 2020.[10], [11] Glaucoma stability on treatment is assessed by monitoring the visual field with SAP tests, repeated at intervals of between 2 months and 2 years over a patient's lifetime. Computational methods are required to analyse series of SAP data to identify change; without these, even experienced clinicians have been shown to make inconsistent decisions.[12], [13] Current statistical approaches typically use ordinary least squares regression over time to track changes in summary measures, regions of interest or individual visual field locations.[14]–[17] Other methods simply make comparisons between the most recent test(s) and baseline measurements.[18]

Current methods for detecting change in series of DLS measurements are inadequate because they do not sufficiently address the complexity of the data,[19] notably non-stationary variability and spatial correlation. SAP measurements of retinal function are indirect because of the psychophysical processes involved – a person's response depends on the probability of perceiving and responding to a light stimulus (Figure 1d). The consequence is considerable variability that increases as DLS deteriorates with the disease progresses, eventually becoming censored in blind regions.[20]–[22]. For instance, when DLS is healthy at 32 dB, the repeat measurement range (90% confidence interval) is 7 dB (26 dB to 33 dB), while this range increases to 18 dB (5 dB to 27 dB) when the DLS deteriorates to 20 dB. This changing variability over time is referred to as ‘non-stationary measurement variability’. Furthermore, SAP measurements are made in a regular grid across a patient's field of view, but this grid does not respect the anatomical arrangement of the retinal nerve fibres that transmit signals from the retina to the brain (Figure 1c).[23] The division of the grid by retinal nerve fibres results in correlation between spatially-related locations. There are prescriptions for modelling this unique spatial process,[24] but they have yet to be incorporated into analysis of series of SAP measurements over time. Therefore, without taking into account these statistical properties, detection of change in retinal function with current methods is often delayed, or requires more clinic visits than should be necessary.[25]

To address these issues we propose an analytical approach to handle the variability structure in SAP data and also capture the information about the spatial process underpinning changes in the visual field. This new computational method, analysis with non-stationary *Weibull* error regression and spatial enhancement (*ANSWERS*), is designed to accurately identify changes in SAP measurements acquired over time (Figure 1f). Further, the method can be adapted to investigations of new therapies, so that changes before and after an intervention can be detected. In this study we applied the technique to large scale clinical data sampled from more than 75,000 patients in electronic health records. Specifically, we examine the hypothesis that *ANSWERS* can detect change in retinal function more rapidly than widely used methods based on ordinary least squares linear regression.

## Materials and Methods

### Ethics statement

Patients' data was anonymised prior to investigation and did not contain personal or sensitive information. It was held in a secure database held at City University London. As such patients' written consent for their data to be used in the study was not required. The study adhered to the tenets of the Declaration of Helsinki and was approved by the research governance committee of City University London, United Kingdom. The anonymised dataset can be accessed upon request.

### Datasets

All visual fields were measured via SAP with the Humphrey Field Analyzer (Carl Zeiss Meditec, CA, USA) using the 24-2 test pattern (Figure 1c) and the SITA (Swedish Interactive Thresholding Algorithm) Standard testing algorithm. The test measures retinal DLS at about 50 test locations, where each test location is evenly separated by an angular distance of 6° across the visual field (Figure 1c).

Two datasets collected at different centres were used in this study. The first dataset was sampled from 402,357 visual fields of 75,857 patients from electronic health records of glaucoma clinics at Moorfields Eye Hospital in London. DLS deteriorates as a result of ageing, and typically do not increase in response to standard medical treatments for glaucoma. Thus, all series in the dataset should be worsening at a rate at least equal to age-related decline. When positive rates are observed, in the case of glaucoma, this is usually due to ‘learning effects’ (patients learn to perform the visual field test) or the inherent variability of the measurement. Therefore, the first visual field of each series was discarded to reduce the impact of ‘learning effects’.[26], [27] If multiple visual fields were taken on the same day, the last measurement was chosen. Only series that were obtained over 6 years and contained at least 7 visual fields were included in the study. Note that the length of series is purely for evaluation purposes and is not necessitated by the proposed model. All series meeting the above criterion were selected for this study and the resulting dataset consisted of 47,483 visual field tests from 6,011 series from 6,011 eyes, representing about 2.5 million individual DLS measurements. The median (interquartile range [IQR]) time of follow up was 9.3 (7.9, 10.4) years and the median (IQR) number of visual fields in each time series was 9 (8, 11). The median (IQR) interval between visual field tests was 1.0 (0.6, 1.4) years.

The second dataset was from a study examining the ‘test-retest’ variability of SAP conducted at Dalhousie University, Halifax Canada in a cohort of glaucoma patients. Changes in retinal function are slow in glaucoma. By taking repeat measurements in a short period of time, it is possible to estimate measurement test variability, under the assumption that no measurable deterioration can occur over the observation period.[20] One eye of 30 patients was tested 12 times over a short period (maximum 8 weeks), during which no measureable deterioration may happen. The variance among visual fields in these repeat measures indicates the inherent measurement variability. Furthermore, each of these visual field series, and the same series with arbitrary reordering, represents a ‘stable’ series with no underlying deterioration. The use of randomly reordered series for estimates of measurement variability is an established method used in various studies.[28], [29]

### Computational model

#### Modelling measurement variability with a mixture of *Weibull* distributions

The variability of individual DLS measurements can be estimated by repeating visual field tests in a short period of time.[20] The test-retest dataset consisting of 1980 (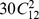, i.e. 30 multiplied by 12-choose-2 combinations) pairs of repeated visual field tests was used to estimate the retest distributions for DLS measurements ranging from 0 dB to 35 dB. Retest distributions are generally bimodal, truncated and skewed; the shape of the distribution varies dramatically across the range of DLS measurements due to the non-stationary variability and the censored nature of the DLS measurement.[22] As the retest distribution could not be sufficiently described by a single parametric probability density function, at each integer level of DLS, it was modelled as a mixture of *Weibull* distributions. The *Weibull* distribution was chosen due to its versatility and relative simplicity. In comparison with commonly used Gaussian distribution, it is a more proper option for modelling probability distribution of non-negative variables like DLS. Its probability density function is defined by two parameters, *α* and *β*:



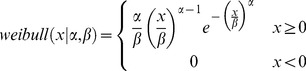
(1)


For *K Weibull* mixture components and *N* retest data points 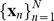, a latent *K*-dimensional binary vector variable 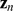 defines to which mixture component the data point 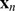 belongs. The *k*th element 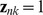 if 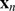 belongs to the *k*th component, otherwise 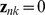. With the prior probability,



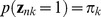
(2)the complete likelihood of observed and latent variables becomes:



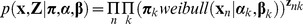
(3)where 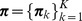 with 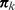 being the prior probability that 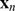 belongs to the *k*th mixture component so 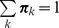. 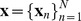, 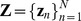, 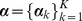 and 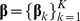 where 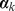 and 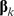 are the parameters defining the *k*th *Weibull* mixture component. Marginalising (3) over 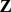 gives the likelihood of *Weibull* mixture distribution:



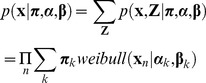
(4)


The maximisation of (4) does not give closed solution for parameters 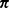, 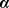 and 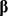. Therefore, an expectation-maximisation algorithm [30] was derived to iteratively optimise (4). The detailed model derivation is given in Appendix S1. Moreover, to select the number of mixture components, *K* was increased from 1 until the logarithm of likelihood in (4) no longer increases with statistical significance (p<1%) in cross validations.

Further, since the log *Weibull* distribution for the minimum DLS in visual field testing, 0 dB, is undefined, a DLS 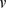 was transformed to 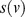 such that:



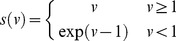
(5)


Note that 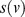 is 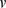 itself except when 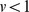 and the lower bound for 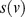 is 0 dB. This transformation guarantees that the transformed DLS 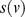 is continuous and has a first derivative, which is an important property for the optimisation of the regression model described in the next section.

For notational simplicity, the derived retest distribution (4) for DLS *y* will hereafter be denoted as 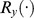.

#### Analysis with non-stationary *Weibull* error regression and spatial enhancement (*ANSWERS*)

We propose a method to monitor change in measurement series, named *ANSWERS*. The proposed model is based on the mixture of *Weibull* retest distributions outlined above, and incorporates spatial correlation within the data.

Given *Q* visual field measurements (each with *M* test locations) in a time series at time 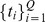, 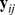 represents a measurement at time 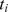 and location *j*. To formulate the regression model in a compact notation, let 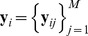, 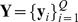, a column vector 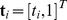 and 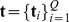.

The regression model is defined by weight vectors 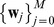 for each of the *M* test locations in the measurement. Each weight vector 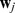 contains a slope and intercept for the *j*th location regressed over time in the case of a linear model. For simplicity of notation, 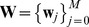 was used to refer to collection of all weight vectors. The likelihood for all visual field measurements in the time series 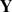 becomes:



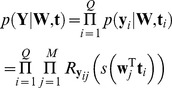
(6)where 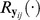 and 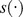 were defined in (4) and (5) respectively. Note that 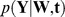 can be factorised into the product of 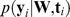 for *i* from 1 to *Q* because 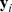 is conditionally independent of other measurements in the series given 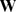.

To incorporate the spatial correlation among different locations in the measurement, prior distributions of slope and intercepts were defined to be multivariate normal distributions:



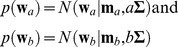
(7)where 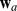 and 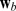 are the slopes and intercepts of the regression lines respectively. 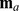 and 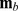 are the means of respective normal distributions and 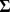 is the covariance matrix scaled by *a* and *b*.

**Figure 2:**
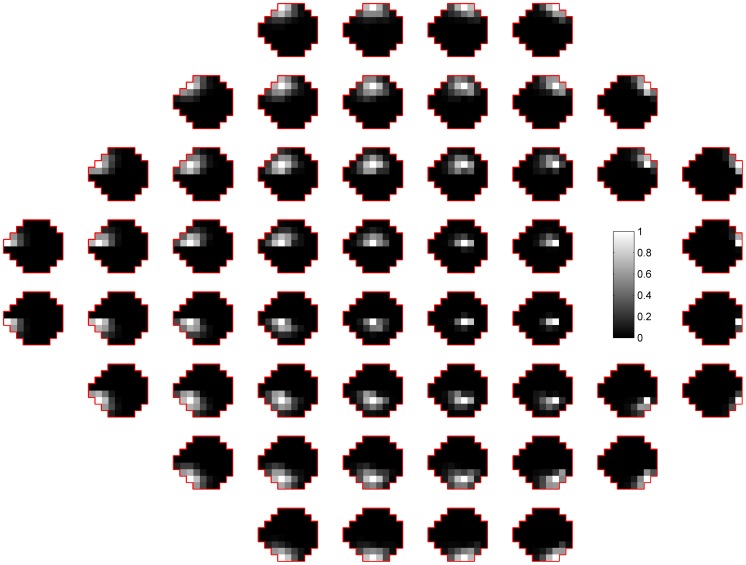
Spatial correlation 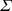 between each location and all other locations in the visual field. The composition of the graph is a 24-2 visual field as shown in Figure 1c. At each visual field location, an image, with the shape of a 24-2 visual field, represents the correlation between this location and all locations in the visual field. The grayscale bar, shown at the location of the blind spot, indicates the level of correlation.

The unscaled covariance matrix 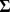 encodes the spatial correlation among test locations in the measurement. The element 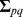 on the *p*th row and *q*th column represents the strength of correlation between points *p* and *q* in the visual field. For visual field DLS measurements investigated in this study, 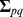 was defined as:



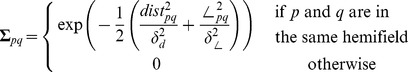
(8)


where 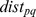 is the Euclidian distance between the points *p* and *q* in the visual field, and 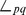 is the difference between the angles that the optic nerve fibres crossing points *p* and *q* enter the optic nerve head.[23], [24] 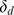 and 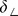 are scale parameters chosen to be 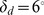 and 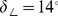. Specifically, 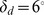 is the distance between two neighbouring points in the visual field and 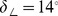 is the reported 95% confidence interval of population variability in the nerve fibre entrance angle into the optic nerve head.[23] Note that 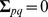 if the two points lie on different hemifields (upper or lower; Figure 1c) of the visual field due to the physiological distribution of optic nerve fibres.[23] The unscaled covariance 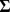 between each location in the visual field and all other points is illustrated in Figure 2. *ANSWERS*, in this study, has been specifically adapted to detect deterioration in glaucoma, so the spatial correlation 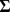 here encodes the anatomy of optic nerve fibres. To adapt *ANSWERS* for other types of measurement corresponding to different diseases or conditions, 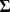 should be adjusted to reflect the characteristics of the spatial correlation present in that data.

The values for the scale parameters *a* and *b* were chosen to produce non-informative priors for 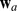 and 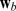. To be exact, the slope prior was set as 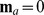
(dB/year) with 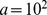 corresponding to a slope standard deviation of 10 dB/year. The intercept prior was set such that 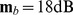 (middle of DLS measurement range) and 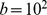
corresponding to an intercept standard deviation of 10 dB.

According to (6) and (7), the log of posterior probability of 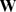 can be derived as:



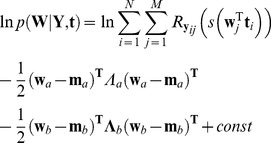
(9)where 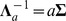, 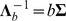. The terms independent of 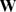 are grouped into the constant term, *const*.

The posterior probability (9) cannot be recognised as a known distribution because (7) is not the conjugate prior of the mixture of *Weibull* distributions. Although the log posterior (9) can still be maximised with regard to 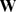, it is difficult to estimate the exact variance of 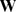 without knowing the underlying distribution. Therefore, a *Laplace* approximation [31], [32] was used to approximate 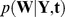 as a normal distribution centred at the mode of 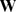, as described in Appendix S1. The estimates of the slope and intercept in the *Laplace* method exactly match the local maximum of log posterior probability (9). However, the variance of these slopes and intercepts are approximate estimates.

For the purpose of evaluating the effects of spatial correlation, its contribution can be ‘switched off’ by setting the off-diagonal elements of 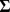 in (7) to be 0. This model without spatial enhancement is denoted as *ANSWER*.

#### *ANSWERS* indices: identification of change

*ANSWERS* estimates the slope 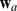 and intercept 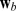 with their variance approximated by the *Laplace* method. The distribution of the slope is of particular clinical importance because it represents the rate and certainty of change. The ‘change’ applies equally to deterioration (negative change) and improvement (positive change) in measurements. In the case of a progressive condition, such as glaucoma, the slope distribution at each location can be summarised as the ‘probability of no-deterioration’, which is quantified as the cumulative distribution of slope ≥0 dB/year. The ‘probability of no-deterioration’ value will be referred to as *Pnd* hereafter. The *Pnd* value ranges between 0 and 1 where a lower value indicates a higher probability of deterioration.

In order to summarise the possibility of deterioration across all *M* test locations in the visual field series, a global index, the *ANSWERS* deterioration index 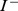, is defined as:



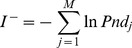
(10)where 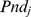 is the *Pnd* value at the *j*th location in the measurement. 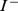 is the negative logarithm of the product of all *Pnd* values, thus, non-negative and larger value implies greater certainty about deterioration in the measurement series. Similarly, to evaluate the improvement in a measurement series, such as in the case of gene therapy for retinal disease,[3] the *ANSWERS* improvement index 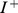 can be derived as 
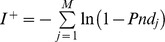
. However, because this study illustrates the application on identifying deterioration in retinal function in glaucoma, the *ANSWERS* index will henceforth only refer to 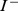.

### Evaluation

To evaluate the utility of *ANSWERS* in detecting retinal function change, it is necessary to compare it to other change detection methods currently used in clinical decision making. Point-wise linear regression, the most widely used method, fits an ordinary linear regression model to a time series of measurement for each location in the visual field and assesses the significance and slope of the fit. Summary measures, such as the mean deviation from the average DLS of healthy eyes, are also utilised, but since glaucoma tends not to affect all locations to the same extent, global indices often have inadequate statistical sensitivity to detect worsening when compared with methods assessing deterioration at individual locations.[14]–[17]. Moreover, to evaluate the benefit of taking into account non-stationary variability and spatial correlation respectively, *ANSWERS* without spatial enhancement (*ANSWER*) was also evaluated. Thus, *ANSWERS* was compared with three other methods: ordinary linear regression of mean deviation, point-wise linear regression and *ANSWER*.

#### Estimation of false positive rates

A false positive is a type I error where change is falsely detected in a series with no true deterioration. The false positive rate can be reliably estimated in a series of repeated measurements acquired in a period of time too short for measureable deterioration. Moreover, randomly reordering these repeated measurements produces pseudo-series where there is also no true deterioration.

The series of 12 visual fields from 30 eyes in the test-retest dataset were randomly reordered 300 times, so 90,000 pseudo-series of length between 3 and 12 were generated. It was assumed that one visual field measurement per year was taken in these pseudo series (the median test frequency in the Moorfields dataset). The false positive rate was then estimated as the proportion of series identified as deteriorating. In a clinical situation, false positives may lead to overtreatment and unnecessary cost, so methods with high false positive rates are generally considered as not clinically useful.

Different methods should be compared at equivalent false positive rates, which is dependent on the chosen change criterion and the length of the series. For ordinary linear regression of mean deviation, deterioration criteria were a negative slope and *p*-value lower than a set threshold. For point-wise linear regression, deterioration criteria for each test location were a negative slope and a *p*-value<1% and the visual field was worsening when at least n = 1, 2, 3 and 4 contiguous points were deteriorating. For *ANSWERS* and *ANSWER*, the criterion for deterioration was a 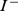 value higher than a given threshold. For each method, a set of thresholds was chosen to achieve specified false positive rates, and the performance of each method was then compared at equivalent false positive rates.

#### Time to detect change

The time to detect deterioration was compared between methods using the dataset from electronic health records at Moorfields Eye Hospital. In each visual field series, a subseries containing the first three visual fields was considered as the minimum series length required to detect change. The length of the subseries was then increased by incrementally adding visual fields to the subseries in chronological order. The shortest series that was flagged as deteriorating was then recorded for each method. If no deterioration was detected in any subseries of a visual field series by a method, the time was recoded as the total time span of the series. The comparison among different methods was carried out at equal false positive rates.

#### Hit rate of change detection

Statistical sensitivity is the measure of a method's ability to identify true change. Ideally, the sensitivity should be evaluated as the proportion of detected change in the visual field series with true underlying deterioration. However, due to the lack of a ‘gold-standard’ and ‘ground-truth’ classification for glaucomatous deterioration,[33] the underlying worsening status of each visual field series was unknown. Therefore, the methods were compared using the ‘hit rate’, which is the proportion of series flagged as deteriorating in the Moorfields dataset. Given an unknown proportion *p*% of truly worsening series in the dataset, the hit rate is linked to statistical sensitivity as: hit rate = (*p*%×sensitivity)+[(1−*p*%)×false positive rate]. Note that if the false positive rate is controlled to be equivalent for all methods, a higher hit rate implies better sensitivity of a method. Therefore, hit rates of all methods were compared as a surrogate comparison for sensitivity.

## Results

*ANSWERS* was implemented in MATLAB R2013a (MathWorks Inc., Natick, MA). Analysis of a series with 10 visual fields took approximately 1.5 seconds on a 2.50 GHz Intel i7 processor. The software is freely available from the authors.

### Mixture of *Weibull* retest distributions

**Figure 3:**
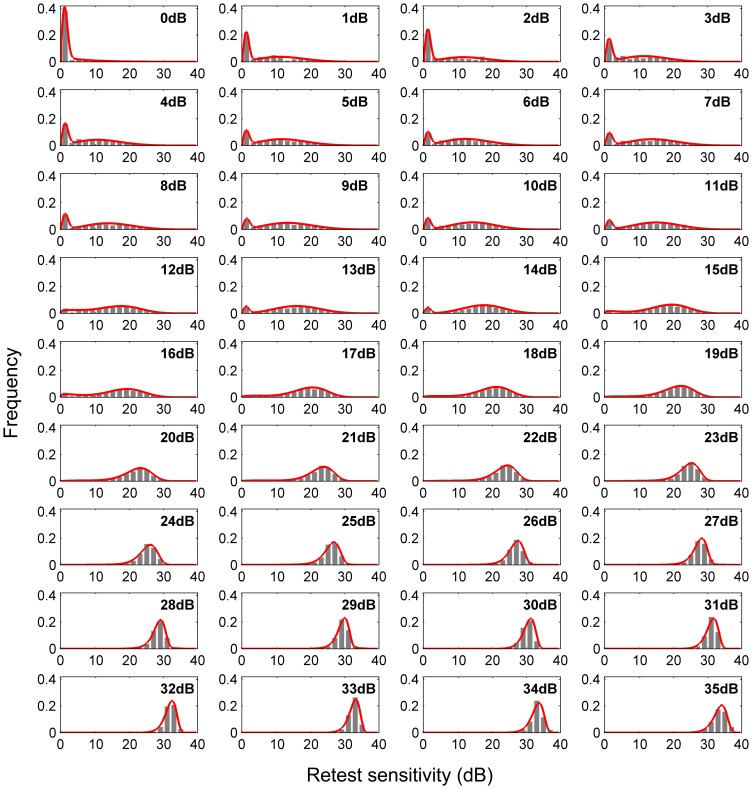
Histograms of retest differential light sensitivities at levels between 0 The derived probability density function of the *Weibull* mixture is superimposed in red.

At all levels of DLS, increasing the number of *Weibull* mixture components to be more than 2 does not significantly increase the log likelihood (4) in cross validations. Therefore, two mixture components were used to model retest distribution for sensitivities between 0 dB and 35 dB. The histograms and the derived probability density functions of the *Weibull* mixture at different DLSs are shown in Figure 3. Despite the non-stationary variability, each distribution can be sufficiently described by a combination of two *Weibull* distributions.

**Figure 4:**
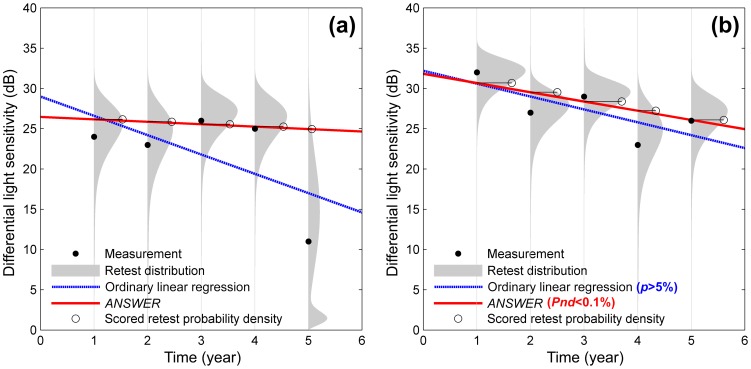
Examples comparing *ANSWER* and ordinary linear regression. The retest distributions of corresponding differential light sensitivity measurements are superimposed as grey areas. The scored probability densities by the *ANSWER* regression line are marked on the retest distributions.

The examples in Figure 4 demonstrate the effect of the *Weibull* mixture retest distribution used by *ANSWER* in comparison to ordinary linear regression in series of DLSs at a single visual field location. Because only a single visual field location was considered for illustrative purposes, there is no spatial enhancement in these examples. In Figure 4a, the last measurement in the series changes suddenly due to measurement variability leading to a steep slope using ordinary linear regression. By comparison, *ANSWER* is less affected by the last measurement since it accounts for the large variability associated with measurements at this level of DLS, and results in a shallower slope. This property of *ANSWER* makes it robust to the non-stationary variability of DLS measurements and, therefore, a more reliable estimator of change rate.

### Time to detect change

Despite the robustness of *ANSWER,* it does not compromise sensitivity to detect deterioration. In fact, by taking into account the non-stationary variability of DLS measurements, the method is able to detect significant deterioration in short time series where conventional methods cannot reach statistical significance. In Figure 4b, ordinary linear regression did not indicate significant deterioration (*p*-value>5%), while *ANSWER* managed to ascertain with high certainty that deterioration was occurring (*Pnd*<0.1%). This property allows *ANSWER* to provide better time efficiency in detecting deterioration.

**Figure 5:**
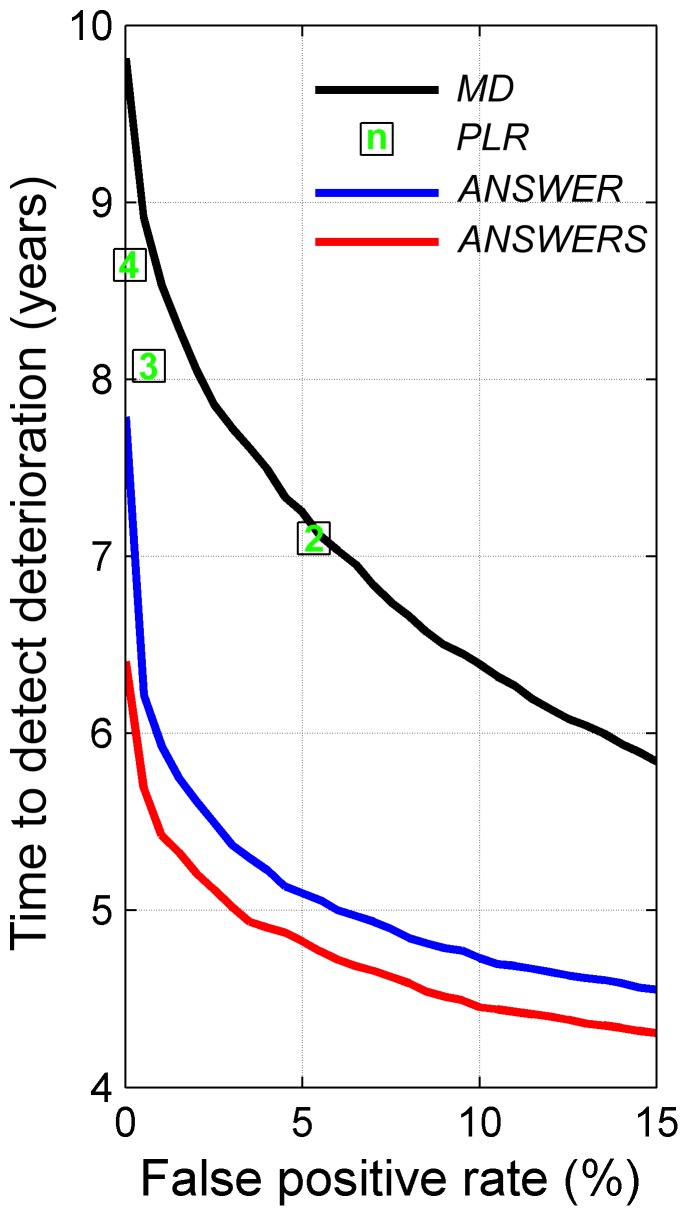
Time to detect deterioration for linear regression of mean deviation (*MD*), point-wise linear regression (*PLR*), *ANSWERS* and *ANSWER* at false positive rates between 0 and 15%. The number of contiguous points in point-wise linear regression are shown in the square points.

Figure 5 shows the average time to first detect deterioration in the visual field series with each method at false positive rates between 0 and 15% (methods with a higher false positive rate are not clinically useful). Because the criteria for point-wise linear regression (the number of contiguous points with deterioration in the visual field) are not continuous, the time efficiency of point-wise linear regression could not be estimated with a continuous false positive rate. Moreover, the false positive rate with the single-point criterion of point-wise linear regression was higher than 15%, so this was not shown in the figure.

For each method, the time to detection change was compared at the 5% false positive rate, or at the closest rate to 5% for point-wise linear regression (two contiguous points, false positive rate of 5.3%). At this false positive rate, *ANSWER* detected deterioration faster than point-wise linear regression (*p*<0.1% paired t-test) and linear regression of mean deviation (*p*<0.1% paired t-test). Furthermore, with spatial enhancement, *ANSWERS* was able to detect deterioration significantly faster than *ANSWER* (*p*<0.1% paired t-test). On average, *ANSWERS* detected deterioration 2.42 (95% confidence interval [2.35, 2.49]) years ahead of point-wise linear regression, 2.28 (95% confidence interval [2.20, 2.35]) years before linear regression of mean deviation, and 0.27 (95% confidence interval [0.22, 0.31]) years before *ANSWER*.

### Hit rate of change detection

**Figure 6:**
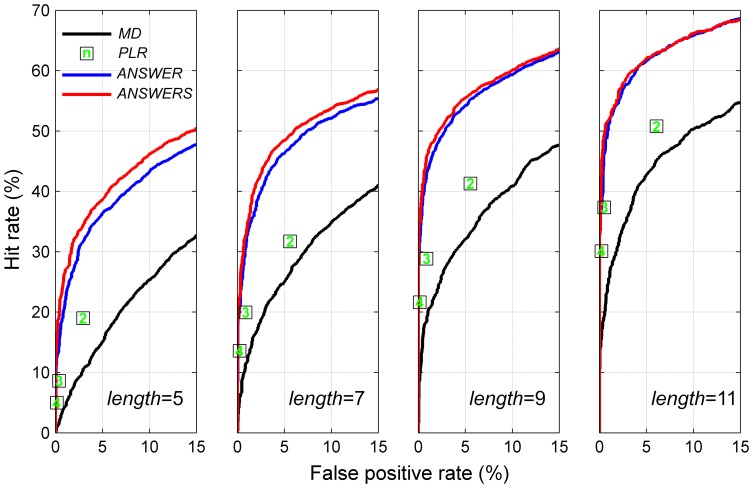
The hit rates of linear regression of mean deviation (*MD*), point-wise linear regression (*PLR*), *ANSWERS* and *ANSWER* with series lengths (*length*) of 5, 7, 9 and 11. The number of contiguous points in point-wise linear regression are shown in the square points. The hit rates are estimated at false positive rates between 0 and 15%.

The hit rates of the four methods were estimated with various series lengths and at false positive rates between 0 and 15% using Moorfields dataset. Figure 6 demonstrates the hit rate with series lengths of 5, 7, 9 and 11. Only the hit rates at specified false positive rates between 0 and 15% are displayed (methods with a higher false positive rate are not clinically useful). The areas under the partial hit rate curves for different methods (Figure 6) were compared in Table 1. Because the total area with false positive rate between 0 and 15% is 0.15, the areas under the partial hit rate curves were normalised by being divided by 0.15. Because the hit rate of point-wise linear regression could not be estimated with a continuous false positive rate, the area under the partial hit rate curve was not estimated.

**Table 1:** The normalised areas under partial hit rate curves for *ANSWER*, *ANSWERS*, linear regression of mean deviation (*MD*).

	Series length = 5	Series length = 7	Series length = 9	Series length = 11
*ANSWER*	0.39	0.48	0.55	0.62
*ANSWERS*	0.41	0.49	0.56	0.62
*MD*	0.20	0.29	0.35	0.44

The comparison was carried out with series lengths of 5, 7, 9 and 11.

The methods were also compared at the 5% false positive rate, or at the closest rate to 5% for point-wise linear regression (two contiguous points criterion). The ratios of hit rates between pairs of methods are shown in Table 2 where a ratio >1 indicates a better hit rate. For instance, with series of 7 visual fields, the ratio of *ANSWERS* against linear regression of mean deviation was 1.9, indicating that the hit rate of *ANSWERS* is nearly twice that of the latter method.

**Table 2:** The ratio of the hit rates for *ANSWER* and *ANSWERS* (in columns) against those of linear regression of mean deviation (*MD*), point-wise linear regression (*PLR*) of differential light sensitivity and *ANSWER* (in rows).

	Series length = 5		Series length = 7		Series length = 9		Series length = 11	
	*ANSWER*	*ANSWERS*	*ANSWER*	*ANSWERS*	*ANSWER*	*ANSWERS*	*ANSWER*	*ANSWERS*
*MD*	2.4, *FP* = 5.0%	2.6, *FP* = 5.0%	1.8, *FP* = 5.0%	1.9, *FP* = 5.0%	1.7, *FP* = 5.0%	1.7, *FP* = 5.0%	1.5, *FP* = 5.0%	1.5, *FP* = 5.0%
*PLR*	1.7, *FP* = 2.9%	1.8, *FP* = 2.9%	1.5, *FP* = 5.6%	1.6, *FP* = 5.6%	1.3, *FP* = 5.5%	1.4, *FP* = 5.5%	1.2, *FP* = 6.1%	1.2, *FP* = 6.1%
*ANSWER*	-	1.1, *FP* = 5.0%	-	1.05, *FP* = 5.0%	-	1.02, *FP* = 5.0%	-	1, *FP* = 5.0%

The false positive rate (*FP*) at which the ratio was estimated is also given. The ratio is calculated for criteria giving 5% false positive rates, or at a false positive rate closest to 5% for point-wise linear regression where the false positive rate cannot be continuously estimated. The comparison was carried out with series lengths of 5, 7, 9 and 11.

The hit rates of *ANSWER* and *ANSWERS* were higher than linear regression of mean deviation and point-wise linear regression of DLS at all series lengths. There was particular improvement in short series. This explains the better efficiency of *ANSWER* and *ANSWERS* to detect deterioration more quickly. The spatial enhancement included in *ANSWERS* also increased the hit rate compared with *ANSWER*, especially with short series. However, this improvement became marginal as the length of series increased.

Case studies with *ANSWERS* in comparison with other methods are provided in Appendix S2.

## Discussion

*ANSWERS* detected change in retinal function more rapidly than conventional statistical approaches without compromising false positive rates. At equivalent false positive rates, it also detected a greater number of eyes with change in retinal function when compared to the number detected by other widely used methods. The *Weibull* mixture retest distributions, in comparison to a normally distributed error assumed in ordinary regression models, allows *ANSWERS* to attain a high certainty about deterioration status (Figure 4b). In addition, the spatial enhancement aggregates information for adjacent locations in the visual field to ‘confirm’ the spatial deterioration pattern, further improving the method especially for short time series. This spatial element of detecting change in visual fields has rarely been considered before.[34]–[37] *ANSWERS* could not only aid clinical decision for prompt treatment intervention, but also define more efficient endpoints for clinical trials in eye-related research.[3]

The application and usefulness of *ANSWERS* in short series is of particular clinical interest. Current widely used methods typified by ordinary linear regression for change detection are limited in short series because they can hardly reach required statistical significance. In clinical situations, where follow-up testing is infrequent, often due to limited resources, these standard analyses may delay the detection of change in retinal function. In turn this can delay required intensification of treatment. In clinical trials, failing to pick up change in time could also lengthen the trials.

When choosing thresholds for *ANSWERS* to detect deterioration in visual field series, it is critical to consider the false positive rate for the chosen threshold of 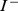. In this study, the threshold was estimated from the test-retest dataset at given false positive rates and for each visual field series length. However, an analytical prescription can be described theoretically and is made available in Appendix S1. Note that 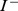 threshold does not change with series length given a constant false positive rate.

The *Laplace* method used in *ANSWERS* provides local normal approximation at the mode of the posterior slope and intercept distribution (9), so estimations of variance of these regression parameters may not capture every feature of the distribution (skewness for example). Although the true posterior distribution (9) is unknown, the estimated slope variance from the *Laplace* approximation was nonetheless demonstrated to be an effective variable in detecting change and quantifying the certainty about change relative to other current methods.

*ANSWERS* was developed with the idea that it could be adapted for other applications with similar statistical properties which are not uncommon among other medical and biological measurements. For example, serum creatinine measurement for predicting kidney failure,[38] heart rate measurement for assessing heart attack risk [39] and baroreceptor sensitivity feedback in diabetes mellitus [40] pose similar challenges in clinical decision making. There are two necessary steps in order to adapt *ANSWERS* for application to other types of clinical measurements. First, the non-stationary variability should be derived from the measurement in question. Since the *Weibull* mixture distribution is versatile and concise, it could easily be adjusted to model other retest distributions using the expectation-maximisation algorithm presented in Appendix S1. Second, the current spatial correlation (8) stems from the anatomy of retinal nerve fibres and therefore is not directly applicable to measurements and conditions other than optic neuropathies. Thus, the spatial correlation in (8) would need to be adapted (or removed if necessary) to reflect the spatial characteristics of the measurement or disease process in question. Moreover, *ANSWERS* was used to infer linear change because there are generally insufficient data to identify non-linear change due to the short visual field series in clinical practice; however, configuring *ANSWERS* to measure change of conditions with long series and temporal processes showing non-linear change is trivial, and can be done by changing the time vector 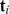 in (6) to nonlinear components such as radial basis functions.

In this study, test-retest data were used to estimate variability and false positive rates. Due to the lack of gold standard about deterioration in retinal function, these data were acquired within a very short period of time (12 visual fields in less than 8 weeks) so it is highly unlikely that measurable damage occurred in this period. However, the patients that make up this dataset may gain psychophysical experience quicker than general clinic patients who typically undertake perimetry tests much less frequently. Therefore patients in the test-retest data could produce measurements with lower variability than that observed in clinical practice. However, all methods were evaluated using the same test-retest data, hence the false positive rates would be equivalently underestimated for each technique. Therefore, despite the potential to underestimate variability, test-retest data does allow us to makes a fair comparison among the methods evaluated.

It is important to note that despite the evolution of new statistical methods for analysing change in retinal function, improving data acquisition techniques should continue to be at the forefront of research. Producing less variable data at the point of measurement acquisition will allow more accurate change detection. Studies have already demonstrated various approaches to improve measurements of DLS. Examples include, but are not limited to, modulation in stimulus size,[41], [42] testing in a linear scale rather than a log scale [43] and increasing the density or changing spatial arrangement of test points.[44], [45] It was also reported that DLS less than 15 dB is not associated with the loss of ganglion cells and may not contain significant information about the integrity of retinal function.[46] Therefore, there is a real need to accurately measure changes in DLS sooner while it exceeds 15 dB.

In conclusion, *ANSWERS* provides a solution in a landscape of uncertainty in detecting retinal function deterioration. This could, for example, impact on how patients with glaucoma are monitored and treated and the efficiency and duration of clinical trials. *ANSWERS* was shown to outperform conventional methods of detecting retinal function deterioration both in terms of statistical sensitivity, and in time taken to detect change. *ANSWERS* was demonstrated to detect visual field deterioration caused by glaucoma, but there is plenty of scope for its use in other measurements subject to non-stationary variability and spatial correlation.

## Supporting Information

Appendix S1 **Detailed mathematical derivation.** Expectation-maximisation algorithm for *Weibull* mixture distribution, Laplace approximation for *ANSWERS* and an analytical model for calculating *ANSWERS* threshold given false positive rates and series lengths.
(PDF)

Appendix S2 **Examples illustrating** ***ANSWERS*** **in comparison with other methods under study.**
(PDF)
