# Supplementary material for: Detecting Changes in Retinal Function: Analysis with Non-Stationary Weibull Error Regression and Spatial Enhancement (ANSWERS)
Source: PLoS One. 2014 Jan 17;9(1):e85654. doi: 10.1371/journal.pone.0085654 (PMC3894992; doi:10.1371/journal.pone.0085654)
Supplement: Appendix S1 — Detailed mathematical derivation. Expectation-maximisation algorithm for Weibull mixture distribution, Laplace approximation for ANSWERS and an analytical model for calculating ANSWERS threshold given false positive rates and series lengths. (PDF) [file pone.0085654.s001.pdf]

Appendix S1

Expectation-maximisation algorithm for mixture of *Weibull* distributions

Similar to typical mixture models, maximising the likelihood (4) does not provide a closed solution for the estimation of $\boldsymbol{\pi}$, $\boldsymbol{\alpha}$ and $\boldsymbol{\beta}$, so an expectation-maximisation (EM) algorithm [1] was used to form an iterative optimisation. The EM algorithm is a widely used parameter estimation technique that starts with initial values of parameters and iterates between the expectation (E-) and maximisation (M-) steps until convergence. Convergence was indicated by an absolute difference of log marginal distribution (4) of less than 10^{-6} between two consecutive iterations.

The EM algorithm was initialised through the selection of starting values for parameters $\boldsymbol{\pi}$, $\boldsymbol{\alpha}$ and $\boldsymbol{\beta}$. $\boldsymbol{\pi}_k = \frac{1}{K}$ and $\boldsymbol{\alpha}$ and $\boldsymbol{\beta}$ were set as random numbers in the range between 5 and 20. In the E-step, the expectation of $\mathbf{z}_{nk}$ under the distribution $p\left(\mathbf{Z} \mid \mathbf{X}, \boldsymbol{\pi}^{old}, \boldsymbol{\alpha}^{old}, \boldsymbol{\beta}^{old}\right)$ was evaluated as:

$$\boldsymbol{\gamma}_{nk} = \mathrm{E}_{p\left(\mathbf{Z}|\mathbf{X},\boldsymbol{\pi}^{old},\boldsymbol{\alpha}^{old},\boldsymbol{\beta}^{old}\right)}\left(\mathbf{z}_{nk}\right) = \frac{\boldsymbol{\pi}_k \, weibull\left(\mathbf{x}_n \mid \boldsymbol{\alpha}_k^{old}, \boldsymbol{\beta}_k^{old}\right)}{\sum_{k'} \boldsymbol{\pi}_{k'} \, weibull\left(\mathbf{x}_n \mid \boldsymbol{\alpha}_{k'}^{old}, \boldsymbol{\beta}_{k'}^{old}\right)} \tag{11}$$

where $\boldsymbol{\alpha}^{old}$ and $\boldsymbol{\beta}^{old}$ are initial *Weibull* distribution parameters if the E-step is in the first iteration, or otherwise are estimated parameters from the previous M-step.

The M-step maximises the expectation of complete log likelihood (3) $\ln p\left(\mathbf{X}, \mathbf{Z} \mid \boldsymbol{\pi}, \boldsymbol{\alpha}, \boldsymbol{\beta}\right)$ under the distribution $p\left(\mathbf{Z} \mid \mathbf{X}, \boldsymbol{\pi}^{old}, \boldsymbol{\alpha}^{old}, \boldsymbol{\beta}^{old}\right)$ with respect to $\boldsymbol{\pi}$, $\boldsymbol{\alpha}$ and $\boldsymbol{\beta}$:

$$\mathrm{E}_{p\left(\mathbf{Z}|\mathbf{X},\boldsymbol{\pi}^{old},\boldsymbol{\alpha}^{old},\boldsymbol{\beta}^{old}\right)}\left(\ln p\left(\mathbf{x}, \mathbf{Z} \mid \boldsymbol{\pi}, \boldsymbol{\alpha}, \boldsymbol{\beta}\right)\right) = \sum_n \sum_k \boldsymbol{\gamma}_{nk}\left(\ln \boldsymbol{\pi}_k + \ln weibull\left(\mathbf{x}_n \mid \boldsymbol{\alpha}_k, \boldsymbol{\beta}_k\right)\right) \tag{12}$$

where $\boldsymbol{\gamma}_{nk}$ was evaluated in (11) by the E-step. Maximizing (12) with respect to $\boldsymbol{\pi}_k$ under the constraint $\sum_k \boldsymbol{\pi}_k = 1$ using a Lagrange multiplier gave:

$$\boldsymbol{\pi}_k = \sum_n \boldsymbol{\gamma}_{nk} \tag{13}$$

Because there is no closed solution for $\boldsymbol{\alpha}$ and $\boldsymbol{\beta}$, the maximisation of (12) with respect to $\boldsymbol{\alpha}$ and $\boldsymbol{\beta}$ was carried out using the Quasi–Newton algorithm.[2,3] This avoids the exact computation of the Hessian matrix of parameters as required by the Newton's method.[3,4] The Quasi–Newton algorithm makes use of the gradient

of (12) with respect to $\boldsymbol{\alpha}$ and $\boldsymbol{\beta}$, which can be derived from (1) and (12). The Quasi–Newton algorithm was set to stop when the absolute change of (12) in two consecutive iterations was lower than 10^{-6}.

Laplace approximation for posterior distribution $p(\mathbf{W} \mid \mathbf{Y}, \mathbf{t})$

A Taylor expansion of (9) was taken around the mode $\mathbf{W}_{\max}$ of (9). For the purpose of optimisation, $\mathbf{W}$ and $\mathbf{W}_{\max}$ were treated as vectors.

$$\begin{aligned} \ln p(\mathbf{W} \mid \mathbf{Y}, \mathbf{t}) \approx \ln p(\mathbf{W}^{\max} \mid \mathbf{Y}, \mathbf{t}) + (\mathbf{W} - \mathbf{W}^{\max})^{\mathbf{T}} \nabla \ln p(\mathbf{W}^{\max} \mid \mathbf{Y}, \mathbf{t}) \\ -\frac{1}{2}(\mathbf{W} - \mathbf{W}^{\max})^{\mathbf{T}} \mathbf{H}(\mathbf{W} - \mathbf{W}^{\max}) \end{aligned} \tag{14}$$

where $\mathbf{H} = -\nabla\nabla \ln p(\mathbf{W} \mid \mathbf{Y}, \mathbf{t})$ is the negative Hessian matrix of (9). Because $\mathbf{W}^{\max}$ is a saddle point of $\ln p(\mathbf{W} \mid \mathbf{Y}, \mathbf{t})$, $\nabla \ln p(\mathbf{W}^{\max} \mid \mathbf{Y}, \mathbf{t}) = 0$ and the second term in (14) becomes 0. In this case, $\ln p(\mathbf{W} \mid \mathbf{Y}, \mathbf{t})$ is approximated as a quadratic function of $\mathbf{W}$, indicating that $p(\mathbf{W} \mid \mathbf{Y}, \mathbf{t})$ approximately follows a normal distribution:

$$p(\mathbf{W} \mid \mathbf{Y}, \mathbf{T}) \approx N(\mathbf{W} \mid \mathbf{W}^{\max}, \mathbf{H}^{-1}) \tag{15}$$

There were two steps in the *Laplace* approximation. First, $\mathbf{W}^{\max}$ of (9) was estimated by a scaled conjugate gradient algorithm.[3] The initial parameters of $\mathbf{W}$ were set such that the initial slopes were $\mathbf{m}_a$ in (7) and the initial intercepts were the median of the DLS measurements at corresponding locations in the series. The scaled conjugate gradient algorithm uses the gradient of (9) derived by differentiation using the chain rule and iteratively searches for $\mathbf{W}^{\max}$, which maximises (9). The algorithm stops when the absolute change of the objective function (9) in two consecutive iterations is less than 10^{-6}. Secondly, the Hessian matrix of (9) at $\mathbf{W}^{\max}$ and the approximated covariance matrix $\mathbf{H}^{-1}$ were calculated.

An analytical model for calculating threshold of I^{-}

To extrapolate beyond the maximum series length of the test-retest dataset, the threshold was modelled as a sigmoidal function of the series length (*length*) with its magnitude modulated by an exponential function of false positive rate (*FP*) between 2% and 20%:

$$threshold = \frac{(m \cdot e^{p \cdot FP} + n \cdot e^{q \cdot FP})}{(1 + e^{-a \cdot length - b})} \tag{16}$$

where *m*, *n*, *p*, *q* are parameters for exponential function modulating the sigmoidal function defined by *a* and *b*. The regression model (16) was optimised by the *Trust-Region* algorithm [5] and provided a very good fit (R^2>0.99). The fitted I^- thresholds from (16) with various false positive rates and series lengths are presented in Figure S1, which can be used as a look-up table to calculate I^- threshold given false positive rate and series length. Note that the I^- thresholds does not change with series length given a constant false positive rate.

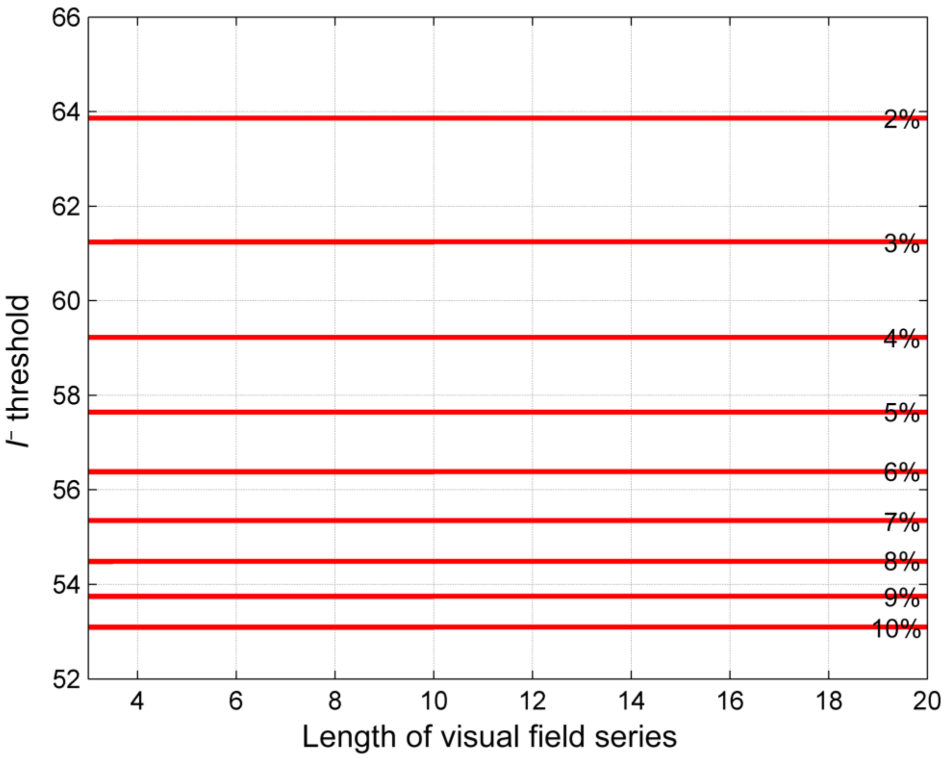

Figure S1. I^- threshold as a function of false positive rate and length of series (number of fields in the series). The I^- threshold was estimated with false positive rates between 2% and 10% and length of series between 3 and 20.

References

1. Dempster A, Laird N, Rubin D (1977) Maximum likelihood from incomplete data via the EM algorithm. Journal of the Royal Statistical Society 39(1): 1–38.

2. Polak E (1971) Computational methods in optimization: a unified approach. New York: Academic Press.

3. Bishop CM (1996) Neural network for pattern recognition. New York: Oxford University Press.

4. Fletcher R (1987) Practical methods of optimization (2nd ed.). New York: John Wiley & Sons.

5. Conn AR, Gould NIM, Toint PL (2000) Trust Region Methods: Society for Industrial and Applied Mathematics.
