# Supplementary material for: Detecting Changes in Retinal Function: Analysis with Non-Stationary Weibull Error Regression and Spatial Enhancement (ANSWERS)
Source: PLoS One. 2014 Jan 17;9(1):e85654. doi: 10.1371/journal.pone.0085654 (PMC3894992; doi:10.1371/journal.pone.0085654)
Supplement: Appendix S2 — Examples illustrating ANSWERS in comparison with other methods under study. (PDF) [file pone.0085654.s002.pdf]

Appendix S2

Figure S2 demonstrates the proficiency of *ANSWERS* at detecting change in a time series with only 3 visual fields. This is for demonstrative purpose and in clinical situation decision should not be solely based on series as short as three measurements. The last three visual fields were simply shown as further evidence of the deterioration status. The linear regression of mean deviation and point-wise linear regression did not reach statistical significance to alert worsening. *ANSWERS*, on the other hand, detected early and rapid deterioration mainly in the superior and the nasal-superior regions of the visual field. This early defect aggressively developed into local blindness in about three years. For the purpose of visualisation, *Pnd* values <5% are indicated in red, suggesting a high likelihood of deterioration. The *ANSWERS* index I^- of 105 is above the threshold corresponding to the 5% false positive rate (56).

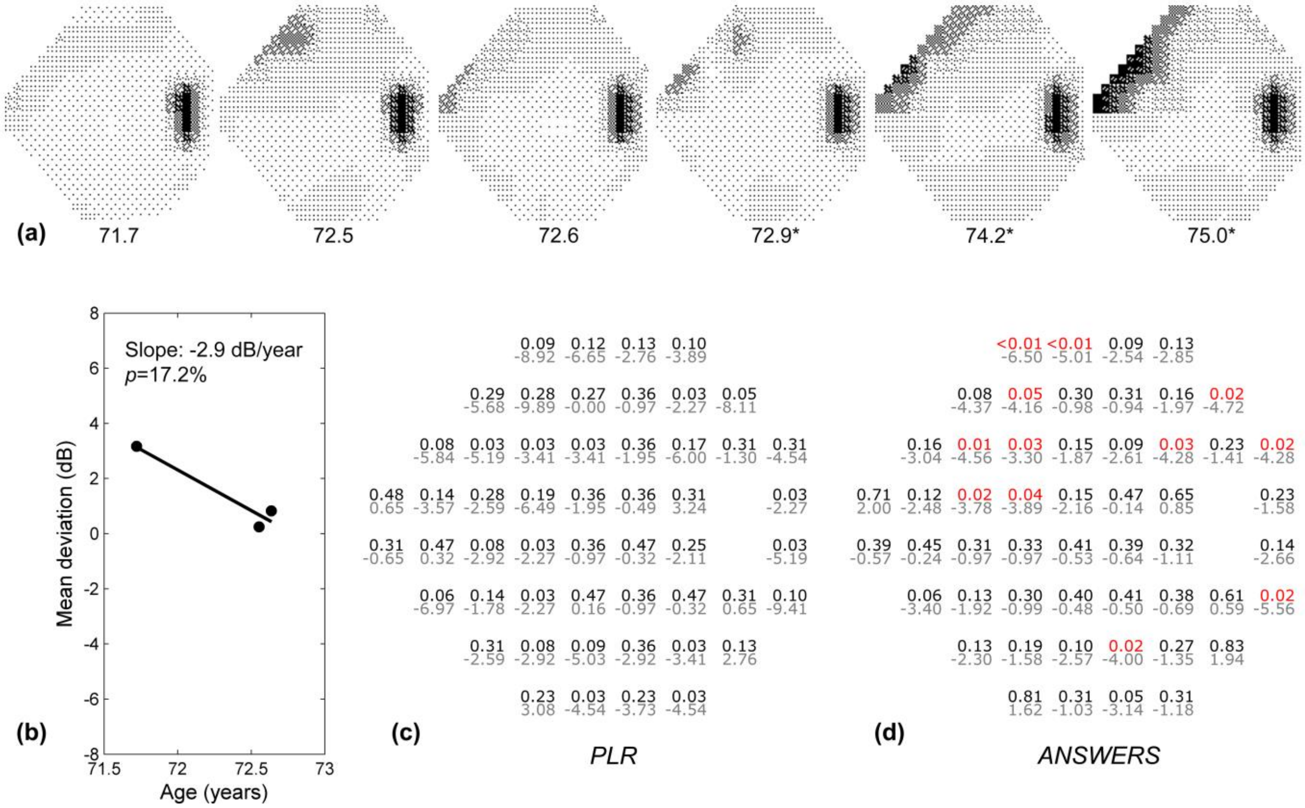

Figure S2. An example of short visual field series. (a) Visual field series in conventional grey scale. The patient's age is shown under each visual field. The visual fields with stars after their age are shown to confirm deterioration status and were not included in the analysis. (b) Linear regression of mean deviation. (c) The *p*-values and slopes in point-wise linear regression (*PLR*). The *p*-values for the locations meeting the point-wise

deterioration criteria (negative slope and *p*-value<1%) are coloured in red. (d) The *Pnd* values and slopes of *ANSWERS*. For visualisation purpose, the *Pnd* values <5% are coloured in red.

DLS deteriorates as a result of ageing, and cannot increase in response to standard medical treatments for glaucoma. Thus, all series in the dataset should be worsening at a rate at least equal to age-related decline. When positive rates are observed, in the case of glaucoma, this is usually due to ‘learning effects’ or the inherent variability of the measurement. This ‘learning effect’ may result in an apparent overall improvement in the visual field, which may mask local deterioration. As an example, Figure S3 shows a visual field series with increasing mean deviation as the patient gains experience with the test. Point-wise linear regression of DLS does not confirm localized deterioration at contiguous points. However, despite an overall apparent improvement in the visual field, *ANSWERS* detects rapid deterioration in the local nasal region and deterioration in the nasal-superior and inferior regions; the *ANSWERS* index I^- of 76 is higher than the threshold at the 5% false positive rate (56).

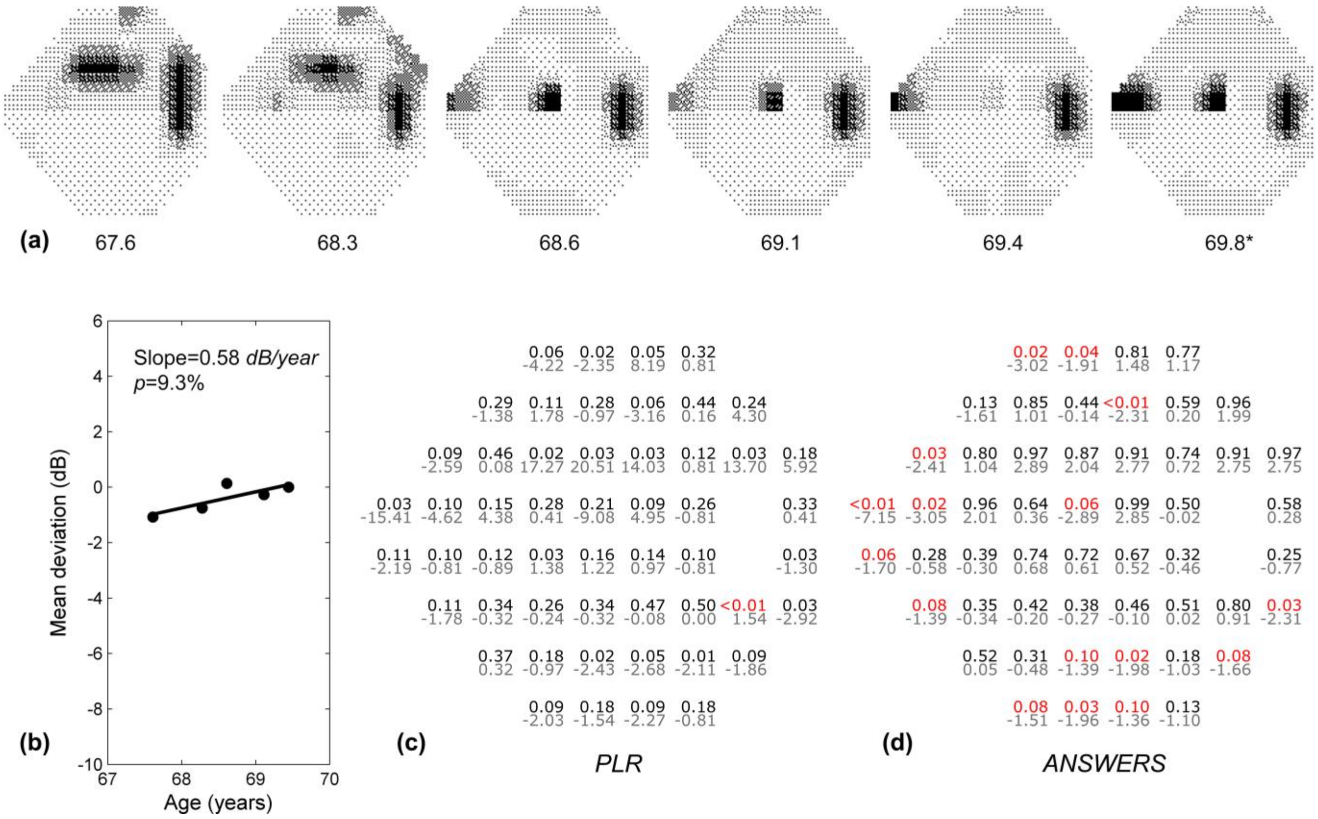

Figure S3. An example visual field series with an improving mean deviation. The figure is arranged in the same configuration of Figure S2.

To demonstrate the benefit of taking into account spatial correlation, especially in short visual field series, an example of *ANSWERS* with and without spatial enhancement is given in Figure S4. *ANSWERS* detects a large number of deteriorating points by aggregating information from adjacent measurements according to their spatial correlation. At the 5% false positive rate, the *ANSWERS* index I^- of 98 was above the threshold of 56, while the I^- of 51 for *ANSWER* was below the threshold of 58.

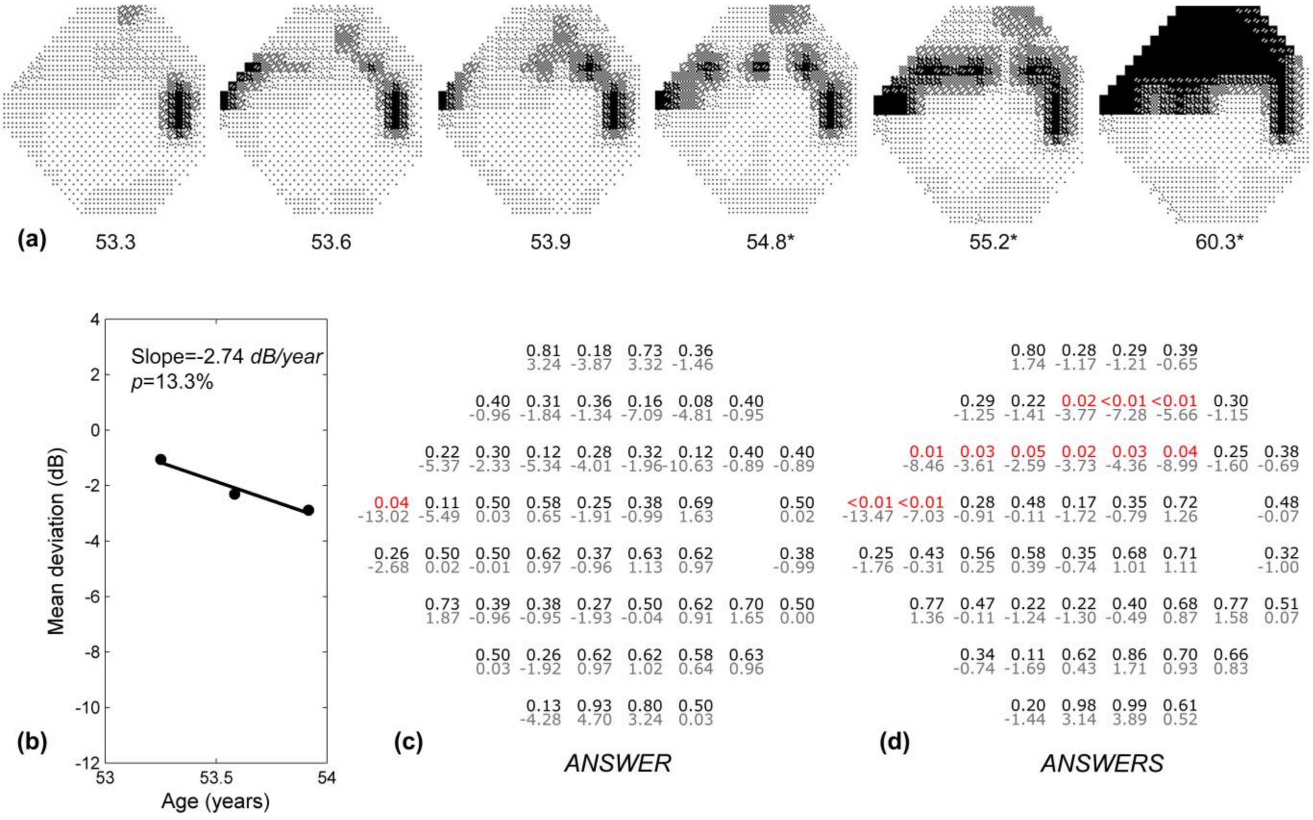

Figure S4. An example visual field series showing the usefulness of spatial enhancement in *ANSWERS*. (a) Visual field series in conventional grey scale. The patient's age is shown under each visual field. The visual fields with stars after their age are shown to confirm deterioration status and were not included in the analysis. (b) Linear regression of mean deviation. (c) The *Pnd* values and slopes of *ANSWERS* without spatial enhancement (*ANSWER*). The *Pnd* values <5% are coloured in red. (d) The *Pnd* values and slopes of *ANSWERS*. The *Pnd* values <5% are coloured in red.
